# Supplementary material for: Hybrid Models and Biological Model Reduction with PyDSTool
Source: PLoS Comput Biol. 2012 Aug 9;8(8):e1002628. doi: 10.1371/journal.pcbi.1002628 (PMC3415397; doi:10.1371/journal.pcbi.1002628)
Supplement: Text S4 — Complete source code for the PyDSTool package (version 0.88.120504). Includes API documentation and help files linking to web pages. This file is identical to the current public release on Sourceforge.net. (ZIP) [file pcbi.1002628.s004.zip › PyDSTool/html/PyDSTool.Toolbox.adjointPRC-pysrc.html]

xml version="1.0" encoding="ascii"?


PyDSTool.Toolbox.adjointPRC


| Home | Trees | Indices | Help | PyDSTool |
| --- | --- | --- | --- | --- |

Package PyDSTool ::
Package Toolbox ::
Module adjointPRC

[hide private]

[frames] | no frames]

# Source Code for Module PyDSTool.Toolbox.adjointPRC

```
  1  from PyDSTool import * 
  2  from PyDSTool.PyCont.misc import getFlowMaps, getFlowJac, getLeftEvecs 
  3   
  4  __all__ = ['adjointPRC', 'rotate_phase'] 
  5   


6 -def adjointPRC(model, limit_cycle, vname, freepar, numIntervals=500, 
  7                 numCollocation=7, method='standard', spike_est=None, 
  8                 saveData=False, doPlot=False, verbosity=0, fname=None, 
  9                 force=False):


10      """Create adjoint PRC from a limit cycle using AUTO, given an 
 11      ODE_Generator or Model object. 
 12   
 13      spike_est (default None) provides an estimate of the spike time in 
 14      the limit cycle, if applicable. If set, this can be useful to 
 15      ensure the PRC is phase-shifted to start at the associated zero 
 16      (in the variable given by vname). 
 17   
 18      method: 'standard' is currently the only working method for calculating 
 19        the eigenvetors corresponding to the unit eigenvalue. 'cyclic' selects 
 20        the cyclic method. 
 21   
 22      Optionally save the PRC data and make plots. 
 23      Returns a dictionary containing the PRC, cycles with independent 
 24      variable values scaled to [0,1], the Jacobian, the flow maps, 
 25      the eigenvector corresponding to the unit eigenvalue, and 
 26      other algorithmic information. 
 27      """ 
 28   
 29      assert isinstance(freepar, str), "Free parameter must be a single string" 
 30   
 31      try: 
 32          MDCont = ContClass(model) 
 33      except: 
 34          print "Problem with model argument" 
 35          raise 
 36   
 37      # If given an LC, use it for continuation 
 38      if limit_cycle is None: 
 39   
 40          PCargs = args(name='EQ1', type='EP-C') 
 41          PCargs.freepars = [freepar] 
 42          PCargs.StepSize = 1e-2 
 43          PCargs.MaxNumPoints = 300 
 44          PCargs.LocBifPoints = 'all' 
 45          PCargs.NumIntervals = numIntervals 
 46          PCargs.NumCollocation = numCollocation 
 47          PCargs.FuncTol = 1e-10 
 48          PCargs.VarTol = 1e-10 
 49          PCargs.SaveEigen = True 
 50          PCargs.SaveFlow = True 
 51          PCargs.SaveJacobian = True 
 52          PCargs.verbosity = 2 
 53          MDCont.newCurve(PCargs) 
 54   
 55          print 'Computing curve...' 
 56          start = clock() 
 57          MDCont['EQ1'].forward() 
 58          print 'done in %.3f seconds!' % (clock()-start) 
 59   
 60          #PCargs.name = 'LC1' 
 61          #PCargs.type = 'LC-C' 
 62          #PCargs.MaxNumPoints = 250 
 63          #PCargs.NumIntervals = 20 
 64          #PCargs.NumCollocation = 4 
 65          #PCargs.initpoint = 'EQ1:H1' 
 66          #PCargs.SolutionMeasures = 'all' 
 67          #PCargs.NumSPOut = 50 
 68          #PCargs.FuncTol = 1e-10 
 69          #PCargs.VarTol = 1e-10 
 70          #PCargs.TestTol = 1e-7 
 71          #PyCont.newCurve(PCargs) 
 72   
 73          # Create bifn curve for limit cycle 
 74          PCargs.name = 'LC1' 
 75          PCargs.type = 'LC-C' 
 76          PCargs.verbosity = verbosity 
 77          #PCargs.freepars = [freepar] 
 78          #PCargs.initcycle = copy(cycle) 
 79          PCargs.initpoint = 'EQ1:H1' 
 80          PCargs.MinStepSize = 8e-3 
 81          PCargs.MaxStepSize = 8e-3 
 82          PCargs.StepSize = 8e-3 
 83          PCargs.MaxNumPoints = 4 
 84          PCargs.LocBifPoints = [] 
 85          PCargs.StopAtPoints = 'B' 
 86          PCargs.NumIntervals = numIntervals 
 87          PCargs.NumCollocation = numCollocation 
 88          PCargs.FuncTol = 1e-10 
 89          PCargs.VarTol = 1e-10 
 90          PCargs.NumSPOut = 3 
 91          PCargs.SolutionMeasures = 'all' 
 92          PCargs.SaveEigen = True 
 93          PCargs.SaveFlow = True 
 94          PCargs.SaveJacobian = True 
 95          MDCont.newCurve(PCargs) 
 96   
 97      # Otherwise, find an LC to use 
 98      else: 
 99          try: 
100              cycle = limit_cycle[MDCont.model.query('variables')] 
101          except: 
102              raise ValueError("Model variables must match cycle coordinate names") 
103          assert vname in cycle.coordnames, "vname argument must be a coordinate" 
104   
105          assert cycle[-1] == cycle[0], "First and last point of cycle must be equal" 
106   
107          assert isinstance(limit_cycle, Pointset), \ 
108                 "cycle argument must be a Pointset" 
109          assert remain(MDCont.model.query('variables'),limit_cycle.coordnames)==[], \ 
110                 "Model variables must match cycle coordinate names" 
111   
112          # Create bifn curve for limit cycle 
113          PCargs = args(name = 'LC1') 
114          PCargs.type = 'LC-C' 
115          PCargs.verbosity = verbosity 
116          PCargs.freepars = [freepar] 
117          PCargs.initcycle = copy(cycle) 
118          PCargs.MinStepSize = 1e-6 
119          PCargs.MaxStepSize = 1e-6 
120          PCargs.StepSize = 1e-6 
121          PCargs.MaxNumPoints = 4 
122          PCargs.LocBifPoints = [] 
123          PCargs.StopAtPoints = 'B' 
124          PCargs.NumIntervals = numIntervals 
125          PCargs.NumCollocation = numCollocation 
126          PCargs.FuncTol = 1e-10 
127          PCargs.VarTol = 1e-10 
128          PCargs.NumSPOut = 3 
129          PCargs.SolutionMeasures = 'all' 
130          PCargs.SaveEigen = True 
131          PCargs.SaveFlow = True 
132          PCargs.SaveJacobian = True 
133          MDCont.newCurve(PCargs) 
134   
135   
136      # Perform continuation to calculate PRC by adjoint method 
137      MDCont['LC1'].forward() 
138      pt = MDCont['LC1'].getSpecialPoint('RG1') 
139      if verbosity > 1: 
140          print "\nRegular point chosen:", pt, "\n" 
141      J = getFlowJac(pt, verbose=verbosity>1) 
142      # n = dimension of system 
143      n = J.shape[0] 
144   
145      if verbosity > 1: 
146          print "Computing flow maps..." 
147      maps, ntst = getFlowMaps(n, pt, 'RG', method=method) 
148   
149      cycle_true = pt.labels['RG']['cycle'] 
150      nint = MDCont['LC1'].NumIntervals 
151      ncol = MDCont['LC1'].NumCollocation 
152   
153      assert isinstance(MDCont.model, NonHybridModel), \ 
154             "Only use a single vector field for the model" 
155      pars = MDCont.model.query('pars') 
156      idxs = ncol*arange(nint) 
157      flow_vecs = array([MDCont.model.Rhs(0, x, pars) for x in \ 
158                         cycle_true[idxs]]) 
159   
160      if verbosity > 1: 
161          print "Computing left eigenvetors corresponding to the unit ", \ 
162            "eigenvalue along flow" 
163      evec1 = getLeftEvecs(n, ntst, maps, flow_vecs, method=method, 
164                           verbose=verbosity>1) 
165      t = cycle_true['t'][idxs] 
166      PRC = Pointset(indepvararray=t/t[-1], 
167                     coordarray=evec1.T/t[-1], 
168                     coordnames=cycle.coordnames) 
169   
170      if spike_est is not None: 
171          assert spike_est >= 0 and spike_est <= t[-1] 
172          spike_est_phase = spike_est*1./cycle_true['t'][-1] 
173          res = PRC.find(spike_est_phase) 
174          if isinstance(res, tuple): 
175              spike_est_ix = res[0] 
176          else: 
177              spike_est_ix = res 
178          prcv = PRC[vname] 
179          phase0_ix = find_zero_phase(prcv, spike_est_ix) 
180          PRC = rotate_phase(PRC, phase0_ix) 
181          phase0_t = PRC['t'][phase0_ix]*t[-1] 
182          if verbosity > 1: 
183              print "spike_est_ix =", spike_est_ix 
184              print "phase0_ix =", phase0_ix 
185              print "phase0_t =", phase0_t 
186          # PRC pointset used a uniform sampling of 
187          # cycle's time mesh points, so get ix in cycle by 
188          # multiplying up phase0_ix by the sampling rate 
189          cycle_true_ph0_ix = phase0_ix*ncol 
190          cycle_true = rotate_phase(cycle_true, cycle_true_ph0_ix) 
191   
192      cycle.indepvararray /= cycle['t'][-1] 
193      T = cycle_true['t'][-1] 
194      cycle_true.indepvararray /= T 
195   
196      if doPlot: 
197          cyc_offset = (max(cycle_true[vname])+min(cycle_true[vname]))/2. 
198          cyc_scale = abs(max(cycle_true[vname])-min(cycle_true[vname])) 
199          PRC_scale = abs(max(PRC[vname])-min(PRC[vname])) 
200          cyc_rescale = PRC_scale/cyc_scale 
201          pylab.figure() 
202          plot(PRC['t'], PRC[vname], 'r', linewidth=2) 
203          plot(cycle_true['t'], cyc_rescale*(cycle_true[vname]-cyc_offset)) 
204          pylab.title('PRC overlay on (cycle%+.2e)*%.2e'%(cyc_offset, 
205                                                    cyc_rescale)) 
206          show() 
207   
208      save_objs = {'starting cycle': cycle, 'PRC cycle': cycle_true, 
209                     'PyCont args': PCargs, 'starting point': pt, 
210                     'PRC': PRC, 'nint': nint, 'ncol': ncol, 
211                     'Jacobian': J, 'maps': maps, 'evec1': evec1, 
212                     'period': T} 
213      if saveData: 
214          # Save the data 
215          if fname is None: 
216              savefile = MDCont.model.name + '_adjointPRC.pkl' 
217          else: 
218              savefile = str(fname) 
219          try: 
220   
221              saveObjects(objlist=save_objs, filename=savefile, force=force) 
222          except ValueError: 
223              print "File already exists -- not saving return objects" 
224   
225      return_objs = save_objs 
226      return_objs['PyCont'] = MDCont 
227   
228      return return_objs

229   
230   


231 -def rotate_phase(pts, phase0_ix):


232      """Phase shift a pointset (assumed to be a cycle) about index 
233      phase0_ix, i.e. 'rotate' it to put the point at phase0_ix at the 
234      beginning of the pointset. 
235   
236      NOTE: Does not update any label info that might be attached to pts! 
237      """ 
238      assert phase0_ix > 0 and phase0_ix < len(pts), "phase 0 index out of range" 
239      try: 
240          t0 = pts['t'][phase0_ix] 
241          parameterized = True 
242      except PyDSTool_KeyError: 
243          parameterized = False 
244      pts_0 = pts[phase0_ix:] 
245      if parameterized: 
246          pts_0.indepvararray -= t0 
247      pts_1 = pts[:phase0_ix] 
248      if parameterized: 
249          pts_1.indepvararray += (pts['t'][-1]-pts['t'][phase0_ix-1]) 
250      pts_0.extend(pts_1) 
251      return pts_0

252   
253   


254 -def find_zero_phase(prc, est_ix):


255      """prc should be of a single variable of interest""" 
256      len_prc = len(prc) 
257      assert est_ix < len(prc) 
258      assert est_ix >= 0 
259      zero_ixs = { 1: len_prc, 
260                  -1:-len_prc}  # out of bounds initial values 
261      for dirn in [1, -1]: 
262          ix = est_ix + dirn 
263          while ix >= 0 and ix < len_prc: 
264              if prc[ix]*prc[est_ix] < 0: 
265                  zero_ixs[dirn] = ix 
266                  break 
267              else: 
268                  ix += dirn 
269      dist_forward = zero_ixs[1] - est_ix 
270      dist_backward = est_ix - zero_ixs[-1] 
271      if dist_forward < len_prc: 
272          phase0_ix = zero_ixs[1] 
273      if dist_backward < dist_forward: 
274          phase0_ix = zero_ixs[-1] 
275      else: 
276          phase0_ix = est_ix 
277      return phase0_ix

278
```

  


| Home | Trees | Indices | Help | PyDSTool |
| --- | --- | --- | --- | --- |

|  |  |
| --- | --- |
| Generated by Epydoc 3.0.1 on Fri May 4 15:24:14 2012 | http://epydoc.sourceforge.net |
